# The diagnostic accuracy of the Patient Health Questionnaire-2 (PHQ-2), Patient Health Questionnaire-8 (PHQ-8), and Patient Health Questionnaire-9 (PHQ-9) for detecting major depression: protocol for a systematic review and individual patient data meta-analyses

**DOI:** 10.1186/2046-4053-3-124

**Published:** 2014-10-27

**Authors:** Brett D Thombs, Andrea Benedetti, Lorie A Kloda, Brooke Levis, Ioana Nicolau, Pim Cuijpers, Simon Gilbody, John P A Ioannidis, Dean McMillan, Scott B Patten, Ian Shrier, Russell J Steele, Roy C Ziegelstein

**Affiliations:** 1 Department of Psychiatry, McGill University, Montreal, Quebec, Canada; 2 Department of Epidemiology, Biostatistics, and Occupational Health, McGill University, Montreal, Quebec, Canada; 3 Department of Medicine, McGill University, Montreal, Quebec, Canada; 4 Department of Educational and Counselling Psychology, McGill University, Montreal, Quebec, Canada; 5 Department of Psychology, McGill University, Montreal, Quebec, Canada; 6 School of Nursing, McGill University, Montreal, Quebec, Canada; 7 Lady Davis Institute for Medical Research, Jewish General Hospital, Montreal, Quebec, Canada; 8 Respiratory Epidemiology and Clinical Research Unit, McGill University Health Centre, Montreal, Quebec, Canada; 9 Library, McGill University, Montreal, Quebec, Canada; 10 Department of Clinical Psychology and EMGO Institute, VU University, Amsterdam, The Netherlands; 11 Psychological Medicine and Health Services Research, Hull York Medical School, York, UK; 12 Department of Health Sciences, University of York, York, UK; 13 Stanford Prevention Research Center, Department of Medicine, Stanford University, Stanford, CA, USA; 14 Department of Health Research and Policy, Stanford School of Medicine, Stanford University, Stanford, CA, USA; 15 Department of Statistics, Stanford University School of Humanities and Sciences, Stanford, CA, USA; 16 Department of Psychiatry, University of Calgary, Calgary, Alberta, Canada; 17 Department of Community Health Sciences, University of Calgary, Calgary, Alberta, Canada; 18 Department of Mathematics and Statistics, McGill University, Montreal, Quebec, Canada; 19 Department of Medicine, Johns Hopkins University School of Medicine, Baltimore, MD, USA; 20 4333 Cote Ste Catherine Road, Montréal, Québec H3T 1E4, Canada

**Keywords:** Patient health questionnaire, PHQ-9, PHQ-8, PHQ-2, Depression, Screening, Diagnostic test accuracy, Systematic review, Individual patient data meta-analysis

## Abstract

**Background:**

Major depressive disorder (MDD) may be present in 10%–20% of patients in medical settings. Routine depression screening is sometimes recommended to improve depression management. However, studies of the diagnostic accuracy of depression screening tools have typically used data-driven, exploratory methods to select optimal cutoffs. Often, these studies report results from a small range of cutoff points around whatever cutoff score is most accurate in that given study. When published data are combined in meta-analyses, estimates of accuracy for different cutoff points may be based on data from different studies, rather than data from all studies for each possible cutoff point. As a result, traditional meta-analyses may generate exaggerated estimates of accuracy. Individual patient data (IPD) meta-analyses can address this problem by synthesizing data from all studies for each cutoff score to obtain diagnostic accuracy estimates. The nine-item Patient Health Questionnaire-9 (PHQ-9) and the shorter PHQ-2 and PHQ-8 are commonly recommended for depression screening. Thus, the primary objectives of our IPD meta-analyses are to determine the diagnostic accuracy of the PHQ-9, PHQ-8, and PHQ-2 to detect MDD among adults across all potentially relevant cutoff scores. Secondary analyses involve assessing accuracy accounting for patient factors that may influence accuracy (age, sex, medical comorbidity).

**Methods/design:**

Data sources will include MEDLINE, MEDLINE In-Process & Other Non-Indexed Citations, PsycINFO, and Web of Science. We will include studies that included a Diagnostic and Statistical Manual or International Classification of Diseases diagnosis of MDD based on a validated structured or semi-structured clinical interview administered within 2 weeks of the administration of the PHQ. Two reviewers will independently screen titles and abstracts, perform full article review, and extract study data. Disagreements will be resolved by consensus. Risk of bias will be assessed with the Quality Assessment of Diagnostic Accuracy Studies-2 tool. Bivariate random-effects meta-analysis will be conducted for the full range of plausible cutoff values.

**Discussion:**

The proposed IPD meta-analyses will allow us to obtain estimates of the diagnostic accuracy of the PHQ-9, PHQ-8, and PHQ-2.

**Systematic review registration:**

PROSPERO CRD42014010673

## Background

Major depressive disorder (MDD) is a chronic, disabling condition that is present in 5%–10% of primary care patients [1,2] and 10%–20% of patients with chronic medical conditions [3]. Unidentified and inadequately treated depression has a major impact on overall health and is a robust indicator of poor prognosis, above and beyond other health risk factors [4]. Globally, depression accounts for more years of healthy life lost than any other medical condition [5-8].

Delivery of depression care, however, is often haphazard, and misdiagnosis is common. Physicians may fail to recognize as many as half of all patients with depression [9], and most patients with depression do not receive minimally adequate care [10,11]. At the same time, there is a high rate of overdiagnosis and overtreatment, and the majority of patients who are treated for depression do not meet diagnostic criteria [12,13]. In specialty medicine settings, rates of depression are even higher than in primary care, but health care teams in specialty settings typically have less specific training on recognizing or treating depression than providers of primary care [3]. Thus, improving depression care is a priority [14].

Routine depression screening, which involves the use of self-report questionnaires to identify patients with unrecognized MDD who have not been identified as at risk for depression, has been proposed as a way to improve depression identification and management [15,16]. Recommendations, policy, and implementation, however, are inconsistent. Prior to 2002, no major guidelines recommended depression screening. Then, in 2002, the United States Preventive Services Task Force (USPSTF) recommended routine depression screening in primary care settings with staff-assisted depression care programs in place to ensure accurate diagnosis, effective treatment, and follow-up [17]. In 2005, the Canadian Task Force on Preventive Health Care (CTFPHC) issued a similar guideline [15], and in 2009, the USPSTF reiterated its recommendation [18,19]. However, the USPSTF recommendation has been criticized as based on speculation that depression screening may benefit patients when high-quality care is provided, but not on direct evidence from randomized controlled trials (RCTs) [20,21]. Consistent with this, in 2010, the UK National Screening Committee determined that there was no evidence that depression screening would reduce the number of patients with depression or improve depression symptoms [22], and the UK National Institute for Health and Clinical Excellence recommended against routine depression screening [2]. In 2013, the CTFPHC reconsidered its earlier guideline and also recommended against screening adults for depression in primary care settings [23]. There are numerous recommendations to screen for depression in speciality medicine settings [24-31], but none is based on evidence of benefit from RCTs, and the wisdom of investing health care resources for screening in these settings without evidence has been questioned [32-36].

In its recent decision to recommend against depression screening in primary care, the CTFPHC concluded that although depression screening may frequently occur in clinical practice, no properly designed RCTs of depression screening have demonstrated benefit or how to successfully implement a depression screening program that would reduce the presence of depression. The Task Force expressed concern that the true diagnostic accuracy of commonly used depression screening tools is poorly understood, that existing evidence may overstate what would occur in actual clinical practice, and that screening without demonstrated screening tool accuracy would likely generate a high rate of false positive screens without improving patient care [23].

In studies on the accuracy of depression screening tools, patient scores on self-report depression symptom questionnaires are compared to diagnostic status (MDD versus no MDD) based on a validated diagnostic interview. Diagnostic accuracy is most commonly described in terms of sensitivity, specificity, positive predictive value, and negative predictive value. In depression screening, sensitivity is the probability that a screening test will correctly identify patients with MDD, whereas specificity is the probability that the test will correctly classify patients without MDD as non-cases [37]. Sensitivity and specificity are generally regarded as intrinsic characteristics of a test and independent of prevalence [38,39]. Studies of diagnostic accuracy typically use receiver operating characteristic (ROC) curve analysis, by which sensitivity and specificity associated with all possible cutoff scores are calculated and plotted [40,41]. From the ROC, an optimal cutoff score is chosen which balances the trade-off between increases in sensitivity and decreases in specificity, and vice versa.

Although sensitivity and specificity are most often reported, predictive values are more clinically relevant. The positive predictive value of a screening tool refers to the probability that a patient with a positive screen will have the condition, whereas negative predictive value is the probability that a negative test accurately rules out the condition. Positive and negative predictive values depend on both test accuracy and prevalence [42]. When screening is done in clinical practice, the relevant information for the health care provider is the probability of the patient with positive (and negative) screens having the corresponding condition (e.g., MDD in depression screening).

There are several important shortcomings in existing evidence on depression screening tool accuracy. Most existing studies have been conducted in samples too small to precisely estimate accuracy and have selectively published accuracy results from high-performing cutoffs, but not from other cutoffs, even when the other cutoffs are considered standard [32,36]. Meta-analyses can overcome problems associated with small sample sizes but are unbiased only if accuracy data for all relevant studies and cutoff scores are included. Some meta-analyses [43,44] have focused on results of primary studies for a standard cutoff, if published, but have substituted data from other cutoffs for large numbers of studies that did not report results for the standard cutoff, presumably because the standard cutoff performed poorly in those studies. Other meta-analyses [45-47] have examined results from multiple cutoffs but have similarly been limited to using published accuracy outcomes for each cutoff. The limitations of this method are highlighted by a 2012 meta-analysis of the Patient Health Questionnaire (PHQ-9) [45], a commonly used screening tool [27,48-51]. In that meta-analysis, estimates of sensitivity actually improved as the cutoff increased (i.e., as more severe symptoms were required for detecting cases), which would be mathematically impossible if complete data from all studies were available and analyzed as a single data set.

In addition to selective reporting, inclusion in diagnostic accuracy studies of patients who are already treated and would not be screened in clinical practice may bias results in primary studies and meta-analyses. One study found that >95% of existing diagnostic accuracy studies of depression screening tools have included already-treated patients, thus exaggerating the estimated number of previously unidentified patients who would be detected and likely biasing estimates of accuracy [16].

Finally, individual studies have sample sizes too small to evaluate individual risk characteristics that may influence diagnostic accuracy, and thus, traditional meta-analyses have not been able to address them either. As described by the CTFPHC [23], accuracy analyses based on individual risk characteristics (e.g., age, sex, medical comorbidity) that likely influence the optimal screening cutoff and accuracy are needed.

Individual patient data (IPD) meta-analysis [52] involves using actual patient data obtained from researchers who conducted primary studies, rather than summary results from published or unpublished study reports. The general approach of an IPD meta-analysis in terms of defining a research question, establishing study inclusion and exclusion criteria, identifying and screening studies, and analyzing data does not differ from a traditional meta-analysis [53]. IPD meta-analyses are resource intensive in that they require substantial time to identify and obtain original data, clarify data-related issues with data providers, and generate a consistent data format across studies [52-54]. When implemented effectively, IPD meta-analyses have particular benefits in addressing limitations in published information or where subgroup analyses are needed, but not possible from study-level data available in original reports, as is the case with depression screening accuracy studies. In the context of evaluating the diagnostic accuracy of depression screening tools, IPD meta-analysis has three major advantages compared to traditional meta-analyses. First, IPD meta-analysis can address bias from the selective publication of well-performing cutoff thresholds from small studies since accuracy can be evaluated across all relevant cutoff scores. Second, IPD meta-analysis allows the exclusion of already-treated patients, for whom the tool would not be used to screen for unidentified depression, as treatment status is often available in primary datasets. Third, IPD meta-analysis with large numbers of patients and large numbers of MDD cases allows the incorporation of individual risk factors for depression (e.g., age, sex, medical comorbidity) and study variables (e.g., study setting, risk of bias factors, funding source) that may influence cutoff selection, accuracy, and clinical decision-making.

The nine-item PHQ-9 [55] and the PHQ-2 [56] and PHQ-8 [57], which are two-item and eight-item subsets of the nine items in the PHQ-9, are commonly recommended for depression screening in clinical and research settings [27,48-51]. Thus, the primary objective is to conduct IPD meta-analyses to determine the diagnostic accuracy of the PHQ-9, PHQ-8, and PHQ-2 to detect MDD among adults. A secondary objective is to assess diagnostic accuracy accounting for age, sex, and medical comorbidity, which may influence accuracy.

## Methods/design

This systematic review has been funded by the Canadian Institutes for Health Research (Funding Reference Number KRS-134297). The protocol has been registered in the PROSPERO prospective register of systematic reviews (CRD42014010673). Ethical approval has been obtained by the Research Ethics Committee of the Jewish General Hospital in Montreal.

Our IPD meta-analysis has been designed and will be conducted in accordance with best-practice standards as elaborated in the Cochrane Handbook for Systematic Reviews of Diagnostic Test Accuracy [58] and other key sources [52,53,59]. Results will be reported in concordance with the Preferred Reporting Items for Systematic Reviews and Meta-Analyses (PRISMA) statement [60,61]. To conduct the meta-analysis, we will seek primary datasets that allow us to compare PHQ-9 scores to MDD or major depressive episode (MDE) diagnostic status. Most primary studies use MDD as the reference standard, but some may use MDE, which is identical with respect to the symptoms of depression but does not exclude patients with psychotic disorders or a history of manic episodes. We will extract the necessary items from the PHQ-9 to also evaluate the briefer PHQ-2 and PHQ-8. In addition, we will include studies with only data from the PHQ-2 or PHQ-8 in those meta-analyses.

### Sources of evidence

Our search strategy was developed by a medical librarian and peer-reviewed by another medical librarian. We will search MEDLINE, MEDLINE In-Process & Other Non-Indexed Citations, PsycINFO, and Web of Science. The MEDLINE search strategy was validated by testing against already-identified publications from preliminary searches (see Appendix 1), and no studies with more than five MDD cases were missed. The strategy was then adapted for PsycINFO and Web of Science. We limited our search strategy to these databases based on research showing that adding other databases (e.g., EMBASE) when the MEDLINE search is highly sensitive does not identify additional eligible studies [62]. The Cochrane Handbook for Systematic Reviews of Diagnostic Test Accuracy [58] suggests combining concepts of the index test and the target conditions, but this was redundant for depression screening tools as these tests are limited to testing for depression. Thus, the search strategy for electronic databases was comprised of two concepts: the index test of interest and studies of screening accuracy. There are no published search hedges designed specifically for mental health screening, but several key articles were consulted in developing search terms [63-65]. Search strategies use a combination of subject headings, when available in the database, as well as keywords anywhere in the record. The search was limited to the year 2000 forward since the PHQ was first published in 2001 [55]. See Appendix 1 for detailed information on searches. To supplement electronic searches, we will search reference lists of all included publications and relevant reviews. In addition, we will conduct a related articles search for included papers indexed in MEDLINE using the PubMed “related articles” search feature. We will also contact researchers who have published on the topic to obtain information about additional, unpublished studies. Search results will be initially uploaded into the citation management database RefWorks (RefWorks, RefWorks-COS, Bethesda, MD, USA) then into the systematic review program DistillerSR (Evidence Partners, Ottawa, Canada). The DistillerSR duplication check function will be used to identify citations retrieved from multiple sources. DistillerSR will be used to store and track search results and to track results of the review process.

**Figure 1:**
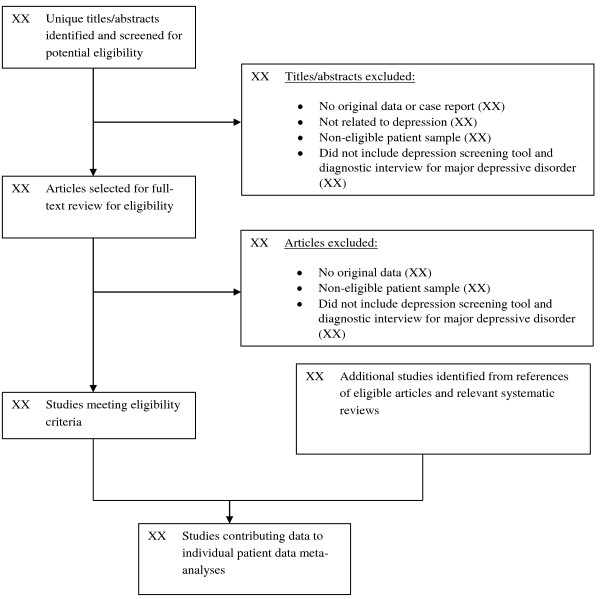
Draft flow diagram of study selection process.

To identify relevant datasets, we will review articles in any language. Datasets will be sought for inclusion if they compare results from the PHQ-9, PHQ-8, or PHQ-2 to Diagnostic and Statistical Manual (DSM) or International Classification of Diseases (ICD) criteria for MDD or MDE. ICD criteria are similar to DSM criteria and generally used outside of North America. Diagnoses must be based on a validated structured or semi-structured interview (e.g., Structured Clinical Interview for DSM [66], Composite International Diagnostic Interview [67]) administered within 2 weeks of the administration of the depression screening tool, since MDD criteria are for symptoms in the last 2 weeks. Datasets where some patients were administered the screening tools within 2 weeks of the diagnostic interview and some patients were not will be included if the original data allows us to select patients administered the diagnostic interview and screening tools within the 2-week window. Data from studies where the PHQ is used with patients already known to have psychiatric diagnoses will be excluded, with the exception of patients treated for substance and alcohol abuse for whom depression screening may be considered. The coding manual for inclusion and exclusion decisions is shown in Appendix 2.Two investigators will independently review titles and abstracts for eligibility. If either reviewer determines that a study may be eligible based on title or abstract review, then a full-text article review will be completed. Disagreement between reviewers after full-text review will be resolved by consensus, including a third investigator as necessary. Chance-corrected agreement between reviewers will be assessed with Cohen’s kappa statistic. Translators will be consulted to evaluate titles/abstracts and articles for languages other than those for which team members are fluent (English, French, Spanish, Dutch, Greek). See Figure 1 for the preliminary PRISMA flow of studies.

### Transfer of data and dataset management

We will contact authors of studies containing datasets that meet our inclusion criteria to invite them to contribute primary data for inclusion.

Per our approved ethics protocol, when an investigator agrees to contribute data, approval for incorporation of the dataset will be sought from the Research Ethics Committee of the Jewish General Hospital in Montreal, which will require documentation of ethics approval and informed patient consent of the primary study. In cases where documentation of the original ethics approval and patient consent forms are not retrievable, ethics approval will be granted if there is other documentation (e.g., publications that document ethics approval and patient consent).

We will ensure that all data that are transferred are properly de-identified prior to transfer. All individual patient data that are obtained will be cleaned and coded to make patient data as uniform as possible across datasets, then entered into a single STATA database (StataCorp, College Station, TX). A preliminary codebook has been developed for coding data from original studies of the PHQ (see Appendix 3). For each study to be included in the dataset, two investigators will independently determine the coding protocol, based on the codebook, with any discrepancies resolved by consensus. Actual data coding and transfer from original studies into the IPD database will be done independently by two supervised staff or trainee members of the team, and resulting datasets will be compared using STATA to identify discrepancies. Data will be stored on password-protected computers with a well-configured firewall, IDS/IPS, up-to-date antivirus and antispyware, passwords for logical access, and a secured backup system.

In addition to obtaining original patient-level data, we will extract data found in the published articles from the studies we included. Two authors will cross-check the published data with the original patient-level data obtained from each dataset, and any inconsistencies will be discussed with the original authors. Corrections will be made as necessary.

### Quality assessment

We will use the Quality Assessment of Diagnostic Accuracy Studies-2 (QUADAS-2) tool [68] to assess risk of bias factors in primary studies, and these factors will be included as study-level variables in analyses. QUADAS-2 incorporates assessments of risk of bias across four core domains: patient selection, the index test, the reference standard, and the flow and timing of assessments. Two reviewers will independently assess risk of bias with any discrepancies resolved by consensus.

### Data analysis

**Figure 2:**
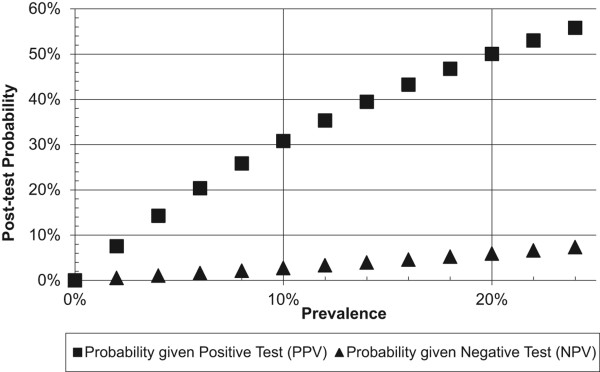
Example of a nomogram depicting post-test probability of major depression as a function of the screening test result for different prevalences and assuming 80% sensitivity and 80% specificity.

Analyses will estimate sensitivity and specificity, which will be used to generate estimates of positive predictive value (PPV) and negative predictive value (NPV), which are more useful clinically. We will fit a bivariate random-effects meta-analysis, estimated via Gaussian Hermite adaptive quadrature, as described in Riley et al. [69], for the full range of plausible cutoff values. This approach models sensitivity and specificity simultaneously and accounts for variation in within-study precision [69]. Data from all primary studies will be analyzed at the same time using a random-effects model so that sensitivity and specificity are assumed to vary across studies. This model will provide us with an overall pooled sensitivity and specificity and an overall pooled diagnostic odds ratio for each cutoff. By combining information across a range of cutoffs, we will be able to construct a pooled ROC curve and identify the cutoff scores [69]. We will present the ROC curves from each primary study, as well as the pooled ROC curve. Optimal cutoffs and the balance between sensitivity and specificity depend on the values of test users [70]. We will identify potentially optimal cutoffs under different scenarios and use these to generate a nomogram, which is a user-friendly graphical depiction of PPV and NPV by prevalence (see example in Figure 2). We will compare results from our IPD meta-analysis to results using only published data. Specifically, we will assess whether optimal cutoffs that maximize combined sensitivity and specificity differ between the two methods. In addition, we will compare sensitivity and specificity estimates across cutoffs using the two methods with deviations of 5% or more considered to be outside an acceptable window of difference.

Heterogeneity will be quantified by reporting the estimated variances of the random effects for sensitivity and specificity, as well as by estimating *R. R* is the ratio of the estimated standard deviation of the pooled sensitivity from the random-effects model to the estimated standard deviation of the pooled sensitivity from the fixed-effects model [71].

In secondary analyses, we will adjust estimates of sensitivity and specificity for age (<60 years versus ≥60 years), sex, and the presence or absence of medical comorbidity. This will allow an estimation of whether the sensitivity and specificity calculated based on the optimal cutoff identified vary according to patient subgroups. Additional study-level covariates may be examined on an exploratory basis. Study-level covariates may include study setting and risk of bias factors described in QUADAS-2. Study setting will initially be delineated as North America or Europe versus from other parts of the world, as well as care setting (e.g., primary care, outpatient specialty care, inpatient care), but may be adjusted based on available data. Administration setting will also be coded (e.g., internet, telephone, in person in acute care setting, in person in outpatient area). QUADAS-2 factors that will be incorporated include patient selection factors, blinding of reference standard to index test results, type of reference standard (e.g., semi-structured diagnostic interview, structured diagnostic interview, physician interview), and timing of administration of index test and reference standard (e.g., same day, delay of 1 to 7 days, delay of >7 days).

Assessing the influence of patient- and study-level factors on diagnostic accuracy can easily be accomplished by including study-, patient- or interaction terms in the random-effects model described above [69]. For patient-level covariates, we will break effects into between-study and within-study components, which is achieved by calculating the study-specific average for the between-study component and the deviation from that average for the within-study component [69,72]. These analyses take advantage of the richness of individual patient data. When analyzed at the patient-level, accounting for correlation between patients from the same study and for the correlation between sensitivity and specificity via the random-effects model, they are more powerful to detect interactions and not vulnerable to ecologic bias compared to traditional meta-analyses [73-76].

To estimate accuracy parameters taking into consideration patient and setting characteristics, we will build predictive models that use the score on the screening questionnaire, as well as age, sex, and other relevant variables to predict MDD. The variables used will be generally available (e.g., age, sex, medical comorbidity, medical setting) and chosen *a priori*, via consultation with specialists from the research team and the literature. The models will be evaluated in terms of their calibration (e.g., slope of linear predictor; are average, low and high predictions correct?) and discrimination (e.g., c-statistic; are low risk subjects distinguished from high risk subjects?) [77]. Validation with the same subjects used to develop a model results in overly optimistic performance. We will assess internal validation via the bootstrap method, which has been shown to be preferable to split sample validation approaches (e.g., developing the model in half the sample and evaluating it in the other half) [78]. Although there are advantages to external validation, given the wide range of study populations that we will be using, it would be unlikely that there would be another comparable dataset large enough for validation. Thus, assessment of internal validity via bootstrapping will allow us to understand how our model will likely perform in a clinical setting, and by using the regression coefficients adjusted for optimism (i.e., the shrinkage estimates), will maximize actual accuracy. Based on our pilot work, we anticipate that missing data will be minimal for the variables of primary interest, and we will impute via multiple imputation using chained equations [77,79], which allow us to impute data for both binary and continuous variables, considering study as a fixed effect in the imputation model [79]. This will allow us to impute both for variables missing for entire studies as well those missing more sporadically.

As sensitivity analyses, we will treat the score as ordinal and use the methods described by Riley et al. to estimate pooled sensitivity and specificity across all thresholds simultaneously [80,81]. In further sensitivity analyses, we will compare studies included in the IPD meta-analysis and those not included in terms of sensitivity and specificity. We will also conduct a sensitivity analysis in which we include aggregate summary estimates of sensitivity and specificity from studies that do not provide individual patient data in our analyses [69].

## Discussion

Depression is a chronic and disabling condition that is the leading global cause of life years lived with disability and plays a major role in coping and prognosis among patients with medical illness [5-8]. However, most patients with depression do not receive adequate care [10,11]. Screening has been proposed as a solution and is currently implemented in practice in many settings in a patchwork fashion. Across national settings, there is a high degree of inconsistency in recommendations, provincial-level policies, and actual practice. There is a need for properly designed, well-conducted trials to determine if depression screening would benefit patients and, if so, to provide models for implementation in clinical practice. Major limitations in existing evidence on the accuracy of depression screening tools, however, present a major barrier to conducting high-quality trials and to potentially including screening as a routine part of clinical practice. The PHQ-9 and briefer versions, the PHQ-2 and PHQ-8, are easily administered, commonly used depression screening tools. By conducting an IPD meta-analyses that address biases in existing evidence and integrates individual patient characteristics into screening, this study will produce an estimate of screening accuracy that is not biased by selective cutoff reporting, that appropriately excludes already-treated patients from the analysis, and that accounts for patient variables that may influence screening accuracy.

## Appendix 1: Search strategies

### MEDLINE (OvidSP)

1. PHQ*.af.

2. patient health questionnaire*.af.

3. 1 or 2

4. Mass Screening/

5. Psychiatric Status Rating Scales/

6. “Predictive Value of Tests”/

7. “Reproducibility of Results”/

8. exp “Sensitivity and Specificity”/

9. Psychometrics/

10. Prevalence/

11. Reference Values/

12. Reference Standards/

13. exp Diagnostic Errors/

14. Mental Disorders/di, pc [Diagnosis, Prevention & Control]

15. Mood Disorders/di, pc [Diagnosis, Prevention & Control]

16. Depressive Disorder/di, pc [Diagnosis, Prevention & Control]

17. Depressive Disorder, Major/di, pc [Diagnosis, Prevention & Control]

18. Depression, Postpartum/di, pc [Diagnosis, Prevention & Control]

19. Depression/di, pc [Diagnosis, Prevention & Control]

20. validation studies.pt.

21. comparative study.pt.

22. screen*.af.

23. prevalence.af.

24. predictive value*.af.

25. detect*.ti.

26. sensitiv*.ti.

27. valid*.ti.

28. revalid*.ti.

29. predict*.ti.

30. accura*.ti.

31. psychometric*.ti.

32. identif*.ti.

33. specificit*.ab.

34. cut?off*.ab.

35. cut* score*.ab.

36. cut?point*.ab.

37. threshold score*.ab.

38. reference standard*.ab.

39. reference test*.ab.

40. index test*.ab.

41. gold standard.ab.

42. or/4-41

43. 3 and 42

44. limit 43 to yr = “2000-Current”

### PsycINFO (OvidSP)

1. PHQ*.af.

2. patient health questionnaire*.af.

3. 1 or 2

4. Diagnosis/

5. Medical Diagnosis/

6. Psychodiagnosis/

7. Misdiagnosis/

8. Screening/

9. Health Screening/

10. Screening Tests/

11. Prediction/

12. Cutting Scores/

13. Psychometrics/

14. Test Validity/

15. screen*.af.

16. predictive value*.af.

17. detect*.ti.

18. sensitiv*.ti.

19. valid*.ti.

20. revalid*.ti.

21. accura*.ti.

22. psychometric*.ti.

23. specificit*.ab.

24. cut?off*.ab.

25. cut* score*.ab.

26. cut?point*.ab.

27. threshold score*.ab.

28. reference standard*.ab.

29. reference test*.ab.

30. index test*.ab.

31. gold standard.ab.

32. or/4-31

33. 3 and 32

34. Limit 33 to “2000 to current”

### Web of Science (Web of Knowledge)

**#1:** TS = (PHQ* OR “Patient Health Questionnaire*”)

#2: TS = (screen* OR prevalence OR “predictive value*” OR detect* OR sensitiv* OR valid* OR revalid* OR predict* OR accura* OR psychometric* OR identif* OR specificit* OR cutoff* OR “cut off*” OR “cut* score*” OR cutpoint* OR “cut point*” OR “threshold score*” OR “reference standard*” OR “reference test*” OR “index test*” OR “gold standard”)

#1 AND #2

Indexes = SCI-EXPANDED, SSCI, A&HCI, CPCI-S, CPCI-SSH Timespan =2000-2014

## Appendix 2: Coding manual

### Title/abstract screening

1. Exclude if no original human data or it is a case study.

Exclude if it is clear from the title and abstract that the article is not an original report of primary data, but for example a letter, editorial, systematic review or meta-analysis, or if it is a case series or single case study. Studies reporting only on animal, cellular, or genetic data are also excluded. Studies that report results in conference abstracts are eligible for inclusion.

2. Exclude if study did not involve administration of the PHQ-9, PHQ-2, or PHQ-8.

Exclude if there is no mention in the title or abstract of any of these versions of the Patient Health Questionnaire. Note that the PHQ-4 (depression and anxiety) includes the PHQ-2.

3. Exclude if there is no assessment of major depression.

Exclude studies if it is clear from the title and abstract that a clinical interview for depression was not conducted. Only studies that assess adults for a DSM diagnosis of MDD or ICD diagnosis of a major depressive episode will be included. Studies that include broader diagnostic categories, such as other depressive (e.g., minor depression, dysthymia) or anxiety disorders, are eligible for inclusion only if they may have separate classifications of adults with MDD or major depressive episode in the primary data. It is unlikely that studies can be excluded at the title/abstract level based on differential diagnosis (e.g., major versus major + minor depression).

4. Exclude if studies do not use a validated diagnostic interview to assess major depression.

Only studies that assess adults for a DSM diagnosis of MDD or ICD diagnosis of a major depressive episode using a validated structured or semi-structured diagnostic interview will be included. Examples of validated diagnostic interviews and other assessment tools that are not validated diagnostic interviews are listed below. Studies that clearly only used a self-report questionnaire to classify patients as depressed are excluded. If studies appear to have conducted a clinical interview to diagnose depression based on the title/abstract review, but it is not clear if a validated diagnostic interview was used, they should be included for full-text review.

*Examples of validated diagnostic interviews*:

Composite International Diagnostic Interview (CIDI)

Diagnostic Interview Schedule (DIS)

Diagnostic Interview Schedule for Children (DISC)

Diagnostisches Interview bei psychischen Störungen im Kindes (Kinder-DIPS)

Mini-International Neuropsychiatric Interview (MINI)

Schedule for Affective Disorders and Schizophrenia (SADS)

Schedules for Clinical Assessment in Neuropsychiatry (SCAN)

Structured Clinical Interview for DSM (SCID)

*Examples of assessment tools that are not validated diagnostic interviews*:

Any self-report measure completed by patients

Hamilton Depression Rating Scale (HAM-D, HDRS)

Montgomery Asberg Depression Rating Scale (MADRS)

Primary Care Evaluation of Mental Disorders (PRIME-MD)

WHO Major Depression Inventory

International Diagnostic Checklist for ICD-10

5. Exclude if PHQ and diagnostic interview are not administered within 2 weeks of each other.

Studies are excluded if it is clear based on the title and abstract that the PHQ and diagnostic interview were not administered within 2 weeks of one another, such as in a longitudinal study that administered one at one time point and the other at a different time point.

6. Exclude if sample selection is based on the presence of distress or depression.

Studies of patients who are pre-selected as possibly distressed or depressed (e.g., based on clinician’s judgment or screening instrument cutoff) prior to administration of the study screening tool and diagnostic interview are excluded. Studies of patients receiving psychiatric treatment or with psychiatric diagnoses are excluded with the exception of studies of substance or alcohol abuse patients. Studies in which only part of the sample is selected based on distress or depression may be eligible if data for patients not selected due to distress levels can be obtained. If only patients above a cutoff score on the PHQ are administered the diagnostic interview, the study is excluded. If, however, a proportion of patients both above and below the PHQ cutoff are administered the interview, the study would be included.

7. Exclude if not adults.

Studies are excluded if it is clear from the title/abstract that the study sample does not include adults aged 18 and over. Studies with mixed population samples are eligible for inclusion if data for adults can be obtained. However, studies that assess only pediatric, adolescent, school, or undergraduate samples will not be included, even if some participants are at least 18 years old.

### Full-text review

1. Exclude if no original human data or it is a case study.

Exclude if the article is not an original report of primary data, but for example a letter, editorial, systematic review or meta-analysis, or it is a case series or single case study. Studies reporting only on animal, cellular, or genetic data are also excluded. Studies that report results in conference abstracts are eligible for inclusion.

2. Exclude if study did not involve administration of the PHQ-9, PHQ-2, or PHQ-8.

3. Exclude if there is no assessment of major depression.

Exclude if patients were not administered the PHQ-9, PHQ-2, or PHQ-8. Note that the PHQ-4 (depression and anxiety) includes the PHQ-2.

Exclude studies if there is not a clinical interview to diagnose MDD based on DSM or a major depressive episode based on ICD. Studies that include broader diagnostic categories, such as other depressive (e.g., minor depression, dysthymia) or anxiety disorders, are eligible for inclusion only if they have classified adults with MDD or major depressive episode in the primary data.

*Examples of inclusion/exclusion of different depression diagnoses*:

DSM-IV-TR:

Include: major depression

Exclude: dysthymic disorder, minor depression (at least two depressive symptoms are present for 2 weeks)

ICD-10:

Include: mild, moderate, severe, recurrent depressive episodes

Exclude: recurrent brief depressive disorder (requires a depressive episode with symptomatic criteria, but lasting less than 2 weeks, and requires that the episodes occur at least once per month for 12 consecutive months)

Research Diagnostic Criteria (RDC):

Include: major depressive disorder

DSM-III:

Include: major depression

Exclude: dysthymic disorder, atypical affective disorders

4. Exclude if studies do not use a validated diagnostic interview to assess major depression.

Only studies that assess adults for a DSM diagnosis of MDD or ICD diagnosis of a major depressive episode using a validated structured or semi-structured diagnostic interview will be included. Examples of validated diagnostic interviews and other assessment tools that are not validated diagnostic interviews are listed below. Studies that clearly only used a self-report questionnaire to classify patients as depressed are excluded.

*Examples of validated diagnostic interviews*:

Composite International Diagnostic Interview (CIDI)

Diagnostic Interview Schedule (DIS)

Diagnostic Interview Schedule for Children (DISC)

Diagnostisches Interview bei psychischen Störungen im Kindes (Kinder-DIPS)

Mini-International Neuropsychiatric Interview (MINI)

Schedule for Affective Disorders and Schizophrenia (SADS)

Schedules for Clinical Assessment in Neuropsychiatry (SCAN)

Structured Clinical Interview for DSM (SCID)

*Examples of assessment tools that are not validated diagnostic interviews*:

Any self-report measure completed by patients

Hamilton Depression Rating Scale (HAM-D, HDRS)

Montgomery Asberg Depression Rating Scale (MADRS)

Primary Care Evaluation of Mental Disorders (PRIME-MD)

International Diagnostic Checklist for ICD-10

5. Exclude if PHQ and diagnostic interview are not administered within 2 weeks of each other.

Studies are excluded if the PHQ and diagnostic interview were not administered within 2 weeks of one another. Datasets where some patients were administered the screening tools within 2 weeks of the diagnostic interview and some patients were not will be included if the original data allows us to select patients administered the diagnostic interview and screening tools within the 2-week window.

6. Exclude if sample selection is based on the presence of distress or depression.

Studies of patients who are pre-selected as possibly distressed or depressed (e.g., based on clinician’s judgment or screening instrument cutoff) prior to administration of the study screening tool and diagnostic interview are excluded. Studies of patients receiving psychiatric treatment or with psychiatric diagnoses are excluded with the exception of studies of substance or alcohol abuse patients. Studies in which only part of the sample is selected based on distress or depression may be eligible if data for patients not selected due to distress levels can be obtained. If only patients above a cutoff score on the PHQ are administered the diagnostic interview, the study is excluded. If, however, a proportion of patients both above and below the PHQ cutoff are administered the interview, the study would be included.

7. Exclude if not adults.

Studies are excluded if the study sample does not include adults aged 18 and over. Studies with mixed population samples are eligible for inclusion if data for adults can be obtained. However, studies that assess only pediatric, adolescent, school, or undergraduate samples will not be included, even if some participants are at least 18 years old.

## Appendix 3: Preliminary individual patient data codebook

### Instructions for each dataset to be included in the IPD Database

1. Save the original database as whatever it was called when it was sent to us and add “(original file)” to the end.

2. Save a new copy of the database as AUTHOR_WITH_ID

••In this file, create a new variable called PT_ID.

••If the database already includes a variable with patient IDs, sort this in ascending order, and then for the new variable, sequentially number the patients, starting with the number 1.

••If the database does not already have a variable with patient IDs, keep the file ordered as is, and then for the new variable, sequentially number the patients, starting with the number 1.

3. Save a new copy of the AUTHOR_WITH_ID database as AUTHOR_IPD.

**Table 1:** Codebook

**Variable**	**Description**	**Values/labels**
STUDY_ID	This variable identifies the database that the data come from, using the primary author or principal investigator of the original study as the label	1 = Study 1
		2 = Study 2
		3 = Study 3
		4 ….
COUNTRY	Country where the study took place	
CLINICAL SETTING	Clinical setting where the study took place	1 = Primary care
		2 = Specialty care
		3 = Non-medical setting
DEPRESSD_PT_ID	DEPRESSD Registry ID.	Sequential numbers from 1 to *n*
AGE	Patient’s age	Numerical value
SEX	Patient’s sex	1 = Female
		2 = Male
		999 = Missing
PHQ9_Q1	Patient data for the first PHQ-9 item: *Interest/pleasure*	0 = Not at all
		1 = Several days
		2 = More than half the days
		3 = Nearly every day
		999 = Missing
PHQ9_Q2	Patient data for the second PHQ-9 item: *Down/depressed/hopeless*	0 = Not at all
		1 = Several days
		2 = More than half the days
		3 = Nearly every day
		999 = Missing
PHQ9_Q3	Patient data for the third PHQ-9 item: *Sleep*	0 = Not at all
		1 = Several days
		2 = More than half the days
		3 = Nearly every day
		999 = Missing
PHQ9_Q4	Patient data for the fourth PHQ-9 item: *Tired/energy*	0 = Not at all
		1 = Several days
		2 = More than half the days
		3 = Nearly every day
		999 = Missing
PHQ9_Q5	Patient data for the fifth PHQ-9 item: *Appetite*	0 = Not at all
		1 = Several days
		2 = More than half the days
		3 = Nearly every day
		999 = Missing
PHQ9_Q6	Patient data for the sixth PHQ-9 item: *Feeling bad about self*	0 = Not at all
		1 = Several days
		2 = More than half the days
		3 = Nearly every day
		999 = Missing
PHQ9_Q7	Patient data for the seventh PHQ-9 item: *Concentrating*	0 = Not at all
		1 = Several days
		2 = More than half the days
		3 = Nearly every day
		999 = Missing
PHQ9_Q8	Patient data for the eighth PHQ-9 item: *Moving*	0 = Not at all
		1 = Several days
		2 = More than half the days
		3 = Nearly every day
		999 = Missing
PHQ9_Q9	Patient data for the ninth PHQ-9 item: *Deaths/hurting self*	0 = Not at all
		1 = Several days
		2 = More than half the days
		3 = Nearly every day
		999 = Missing
PHQ9_Q10	Patient data for final PHQ-9 question: *how difficult these problems have made life*	0 = Not difficult at all
		1 = Somewhat difficult
		2 = Very difficult
		3 = Extremely difficult
		999 = Missing
PHQ9_TOTAL	Total PHQ-9 score (sum of the nine-item scores)	999 = Missing
DEP_CRITERION	Name of diagnostic interview	1 = SCID-IV
		2 = CIDI
		3 = DIS
		4 = SCAN
		5 = MINI
CLASSIF_SYSTEM	Classification system used to classify patients as depressed or not. Include version of DSM/ICD	1 = ICD-10
		2 = DSM-IV
		3 = DSM-III
MDD_DICHOT	Major depression diagnostic status	0 = no MDD
		1 = MDD
		999 = missing
CUR_PSYC_TX	Currently receiving psychological treatment for depression versus not currently receiving psychological treatment for depression	0 = Not currently receiving psychological treatment for depression
		1 = Currently receiving psychological treatment for depression
		777 = Unknown
		999 = Missing
CUR_PHAR_TX	Currently receiving antidepressant medication versus not currently receiving antidepressant medication	0 = Not currently receiving antidepressant medication
		1 = Currently receiving antidepressant medication
		777 = Unknown
		999 = Missing
CUR_UNSPEC_TX	Currently receiving treatment for depression or not currently receiving treatment for depression (type of treatment not specified)	0 = Not currently receiving unspecified treatment for depression
		1 = Currently receiving unspecified treatment for depression
		777 = Unknown
		999 = Missing
ANY_CUR_TX	Currently receiving any kind of treatment (psychological, pharmacological, or unspecified) for depression or not currently receiving any kind of treatment for depression.	0 = Not currently receiving treatment for depression
		1 = Currently receiving treatment for depression
		777 = Unknown
		999 = Missing
MEDICAL_COMORBIDITY	Primary medical comorbidity or multiple comorbidities	0 = None
		1 = CVD
		2 = Diabetes
		3 = Cancer
		4 = Rheumatic disease
		5 = Substance abuse
		6 = Other
		7 = Multiple
RISK_OF_BIAS_PT_SEL	QUADAS-2: Risk of Bias: Patient Selection	1 = Low
		2 = Unclear
		3 = High
RISK_OF_BIAS_INDEX	QUADAS-2: Risk of Bias: Index Test	1 = Low
		2 = Unclear
		3 = High
RISK_OF_BIAS_REF_STD	QUADAS-2: Risk of Bias: Reference Standard	1 = Low
		2 = Unclear
		3 = High
RISK_OF_BIAS_FLOW	QUADAS-2: Risk of Bias: Flow and Timing	1 = Low
		2 = Unclear
		3 = High
APPLIC_PT_SEL	QUADAS-2: Applicability Concerns: Patient Selection	1 = Low
		2 = Unclear
		3 = High
APPLIC_INDEX	QUADAS-2: Applicability Concerns: Index Test	1 = Low
		2 = Unclear
		3 = High
APPLIC_REF_STD	QUADAS-2: Applicability Concerns: Reference Standard	1 = Low
		2 = Unclear
		3 = High

••In this file, transform, recode, and create new variables according to the following codebook (Table 1).

## Abbreviations

CTFPHC: Canadian Task Force on Preventive Health Care; DSM: Diagnostic and Statistical Manual; ICD: International Classification of Diseases; IPD: Individual patient data; MDD: Major depressive disorder; PHQ-2: Patient Health Questionnaire (two-item version); PHQ-8: Patient Health Questionnaire (eight-item version); PHQ-9: Patient Health Questionnaire (nine-item version); PRISMA: Preferred Reporting Items for Systematic Reviews and Meta-analyses; QUADAS-2: Quality Assessment of Diagnostic Accuracy Studies-2; RCT: Randomized controlled trial; ROC: Receiver operator curve; USPSTF: United States Preventive Services Task Force.

## Competing interests

The authors declare that they have no competing interests.

## Authors’ contributions

BDT, AB, LAK, BL, IN, PC, SG, JPAI, DM, SP, IS, RJS, and RCZ contributed to the conception and design of the systematic review and meta-analysis. BDT, LAK, BL, and IN were involved in the acquisition of data. AB and BL analyzed the data. BDT, AB, LAK, BL, IN, PC, SG, JPAI, DM, SP, IS, RJS, and RCZ interpreted the results. BDT and AB drafted this protocol. All authors provided critical revisions of the protocol and approved submission of the final manuscript.
